# Interactions between contaminants and the trophic ecology of two seabirds in a coastal lagoon of the Gulf of California

**DOI:** 10.1007/s10646-025-02853-4

**Published:** 2025-01-13

**Authors:** José Alfredo Castillo-Guerrero, Erick González-Medina, Alberto Piña-Ortiz, Miguel Betancourt-Lozano, Jaqueline García-Hernández, Salvador Hernández-Vázquez, Guillermo Fernández

**Affiliations:** 1 Departamento de Estudios para el Desarrollo Sustentable de Zonas Costeras, Centro Universitario de la Costa Sur, Universidad de Guadalajara, San Patricio-Melaque, México; 2 Conservation Biology Research Group, Área de Zoología, Universidad de Extremadura, Madrid, España; 3 Department of Animal Ecology and Systematics, Justus Liebig University (JLU), Giessen, Germany; 4 Unidad Mazatlán en Acuicultura y Manejo Ambiental, Centro de Investigación en Alimentación y Desarrollo A.C, Mazatlán, Sinaloa México; 5 Unidad Guaymas en Aseguramiento de Calidad y Aprovechamiento Sustentable de Recursos Naturales. Centro de Investigación en Alimentación y Desarrollo A.C. Guaymas, Sonora, México; 6 Unidad Académica Mazatlán, Instituto de Ciencias del Mar y Limnología, Universidad Nacional Autónoma de México, Mazatlán, Sinaloa México; 7 Present Address: Department of Biodiversity, Ecology and Evolution, Faculty of Biological Sciences, Universidad Complutense de Madrid, Madrid, España

**Keywords:** *Fregata magnificens*, foraging ecology, trace elements, *Leucophaeus atricilla*, organochlorine pesticides, Sinaloa

## Abstract

Monitoring the dynamics of contaminants in ecosystems helps understand their potential effects. Seabirds have been used as biomonitors of marine ecosystems for this purpose. However, exposure and vulnerability to pollutants are understudied in tropical species, and the relationships between various pollutants and the trophic ecology of seabirds are poorly understood. In this study, we quantified mercury (Hg), lead (Pb), cadmium (Cd), and organochlorine pesticide (OC) concentrations in the blood of Laughing Gulls and Magnificent Frigatebirds breeding in Bahía Santa María, México. Using carbon and nitrogen isotopic ratios (*δ*^13^C and *δ*^15^N), we examined the interaction between contaminants and trophic ecology. Laughing Gulls exhibited higher concentrations of dichlorodiphenyltrichloroethane and its metabolites (ΣDDTs), endrins (ΣDrins), and chlordanes, while Magnificent Frigatebirds had elevated levels of Hg and hexachlorocyclohexane isomers (ΣHCHs). Both species displayed temporal and sex-related variations in isotopic signatures. Some blood pollutant concentrations in Laughing Gulls were explained by diet: ΣOCs in plasma were directly related to trophic levels, indicating biomagnification, whereas higher Hg levels were associated with changes in habitat use. In contrast, the differences in sex-related isotopic signatures in Magnificent Frigatebirds did not reflect pollutant accumulation patterns, possibly due to their opportunistic feeding habits.

## Introduction

In recent decades, persistent organic pollutants and non-essential trace elements in natural environments have raised notable concerns due to their high toxicity, potential to bioaccumulate in living organisms, and tendency to transfer among trophic levels while persisting in food webs (e.g., Morel et al. 1998; Harrad 2009; Wu et al. 2016; Alharbi et al. 2018). Although most of these pollutants enter the environment through human activities, some are of natural origin, specifically trace elements. Persistent organic pollutants and non-essential trace elements adversely affect organisms by inducing neurotoxic, reproductive, teratogenic, immunological, genotoxic, and tumorigenic effects and organ dysfunction (Ribeiro et al. 2000, 2002; Jayaraj et al. 2016). These effects can compromise survival and long-term reproductive outputs, negatively impacting population dynamics and the functionality of marine ecosystems (Bustnes et al. 2008; Goutte et al. 2014). In particular, coastal and marine environments are highly vulnerable to the accumulation of toxic compounds because they receive large amounts of pollutants from land-based sources, including rivers, drainage ditches, runoff, submarine outfalls, and coastal cities (Sericano et al. 1990; Vikas and Dwarakish 2015).

Some pollutants, such as organochlorines, are highly lipophilic and accumulate in fatty tissues; these pollutants are transferred through the trophic chain and reach their highest levels in top predators (Borgå et al. 2004; Kainz and Fisk 2009). Trace elements do not show a generalized biomagnification pattern, except for mercury, which is transferred as MeHg bonded to sulfur-containing amino acids (Kainz and Fisk 2009). In seabirds, the primary route of exposure to these pollutants is usually feeding (Bearhop et al. 2000; Bocher et al. 2003; Borgå et al. 2004). Therefore, foraging ecology studies are critical to understanding pollutant dynamics and biomagnification. Stable isotope ratios of carbon and nitrogen (*δ*^13^C and *δ*^15^N) are frequently used in trophic studies. The nitrogen isotope ratio (*δ*^15^N) increases with each successive trophic level and is therefore used to estimate the trophic levels of consumers (Bearhop et al. 2002). Indeed, various studies have reported positive relationships between *δ*^15^N (trophic level) and pollutant concentrations, indicating that the uptake of contaminants is directly linked to the food consumed (Jarman et al. 1996; Bearhop et al. 2000; Elliott 2005; Cui et al. 2011). However, some studies have indicated that this pattern is not generalizable and warrants further study (Guzzo et al. 2014; Mello et al. 2016; Costantini et al. 2017). The carbon isotope ratio (*δ*^13^C) increases from inshore to offshore food webs and can be used to determine habitat use (Cherel and Hobson 2007). Moreover, *δ*^13^C fluctuations in the marine environment can be used to distinguish between inshore and offshore sources of contaminants (Peterson and Fry 1987; Kelly 2000), even at large scales. As a result, clear links between foraging areas and contaminant distributions have been previously established (e.g., Ricca et al. 2008; Bond and Diamond 2009). Thus, trends in pollutant dynamics within food webs provide fundamental information on the routes and fates of contaminants in ecosystems (Borgå et al. 2004; Kelly et al. 2007).

Determining seabird diets and their variations can be challenging. However, stable isotope analysis has proven to be particularly effective, as it provides a better understanding of trophic relationships and food sources. By measuring stable isotope values in both seabirds and their prey, researchers can employ mixing models to accurately estimate prey contributions in consumers (e.g., Polito et al. 2011; Karnovsky et al. 2012; González-Medina et al. 2017). This approach can be used to elucidate the feeding habits and ecological interactions of seabirds, which furthers our understanding of their trophic ecology and helps us better understand the transfer of contaminants through food.

Seabirds play crucial roles in the ecological functioning of tropical coastal ecosystems. Several features make seabirds suitable for monitoring marine ecosystems, such as their high sensitivity to environmental change, ease of capture, and the predictability of their spatiotemporal distributions when congregating in colonies (Furness and Camphuysen 1997; Piatt et al. 2007; Elliott and Elliott 2013). However, different seabird species exhibit varying degrees of exposure and vulnerability to pollutants due to differences in diet, foraging strategies, life histories, breeding cycles, physiology, and habitat use (Borgå et al. 2004).

Seabird communities are composed of species with different habitat use strategies and diets, which strongly influence pollutant intake. For example, several studies have indicated that the trophic level (inferred by *δ*^15^N) is directly related with the contaminant load of different tissues (e.g., Carravieri et al. 2014; Sebastiano et al. 2017; Bustamante et al. 2023). In addition, some contaminants exhibit spatial patterns in environmental concentrations (e.g., land-ocean, latitudinal, or vertical), thus the specific habitat use patterns of each species influence the degree of contaminant exposure and accumulation (Sebastiano et al. 2016; Carravieri et al. 2017; Clatterbuck et al. 2021). Moreover, several seabird species show sexual dimorphism, with foraging strategies that may differ between sexes and result in different levels of contaminant exposure and accumulation (e.g., Lerma et al. 2016; Piña-Ortiz et al. 2021; Bustamante et al. 2023). By considering the biological and ecological attributes of several species, contaminant concentrations, and accumulation patterns can be compared to improve our understanding of pollutant exposure and transfer dynamics within ecosystems.

This study aimed to measure the concentrations of non-essential trace elements (Hg, Cd, and Pb) and organochlorine pesticides in the Magnificent Frigatebird and Laughing Gull during the breeding season in Bahía Santa María, Sinaloa, Mexico. We aimed to understand contaminant fluctuations in the blood of these species over time with regard to sex and trophic level. We worked under the premise that a pollution gradient from the coast to the ocean was present, which could be reflected in the seabird species. In general, we expected to find a relationship between the food consumed (isotopic signal) and blood contaminant loads, both among species and at the level of individuals. We expected the Magnificent Frigatebird to occupy a higher trophic level and, thus, exhibit higher bioaccumulation of Hg than the laughing gull. However, considering the coastal and inland spatial use patterns of the laughing gull, this species may be more exposed to some pollutants (e.g., organochlorine pesticides) than the Magnificent Frigatebird. Moreover, considering the feeding habits of both species, we expected higher Cd and Pb concentrations in Laughing Gulls due to their omnivorous diet, which is composed of lower trophic level species, and their use of areas subject to anthropogenic influence (Ceyca et al. 2016; Vizuete et al. 2018). Additionally, as the Magnificent Frigatebird exhibits sexual dimorphism, we anticipated that contaminant accumulation would vary by sex, leading to differential patterns of pollutant accumulation.

## Materials and methods

### Study species

This study focused on two species with different habitat use and sexual dimorphism patterns: Laughing Gull (*Leucophaeus atricilla*) and Magnificent Frigatebird (*Fregata magnificens*). The Laughing Gull is a medium-sized gull (39–46 cm, 216–386 g) that exhibits slight sexual dimorphism, with males being only 1–5% larger than females (Burger 2020). The breeding season of this species lasts from April to July, and both sexes share parental duties; pairs usually care for three-egg clutches and occasionally raise four chicks successfully (Burger 2020). The Laughing Gull forages in offshore, coastal, and inland habitats, usually within 30 km of the colony, although a maximum range of 145 km has been recorded (Dosch, 2003). This species consumes diverse prey, including mollusks, fish, crustaceans, insects, and echinoderms (Burger 2020). The Magnificent Frigatebird is a large and sexually dimorphic seabird (89–114 cm, 1000–1900 g). Females are notably larger and heavier than males (~15% in body mass; Trefry and Diamond 2017). The clutch and brood sizes are invariably one, with both parents caring for the egg and, subsequently, the small chick. However, the male abandons the nest and the colony when the chick is between18 and 160 days old (Osorno 1999), leaving the female to feed the chick for the rest of its time in the nest and the nine-month post-fledging period (Diamond and Schreiber 2020). Due to the extensive duration of parental care, the breeding season of the Magnificent Frigatebird spans the entire year (Diamond and Schreiber 2020). This species forages in coastal and offshore habitats, covering distances of up to 928 km (Austin et al. 2019; Giambalvo et al. 2022). The Magnificent Frigatebird obtains its food primarily by aerial dipping (restricted to a narrow zone measuring a few centimeters immediately above and below the ocean surface; Nelson 1975) or by kleptoparasitizing other seabirds (up to 150 m above land or water; Gibbs and Gibbs 1987). The diet of the Magnificent Frigatebird is mainly composed of flying fish, squid, clupeids, scombroids, discards from prawn-fishing boats, young turtles, crabs, and seabird chicks (Diamond and Schreiber 2020).

### Study area and data collection

**Fig. 1:**
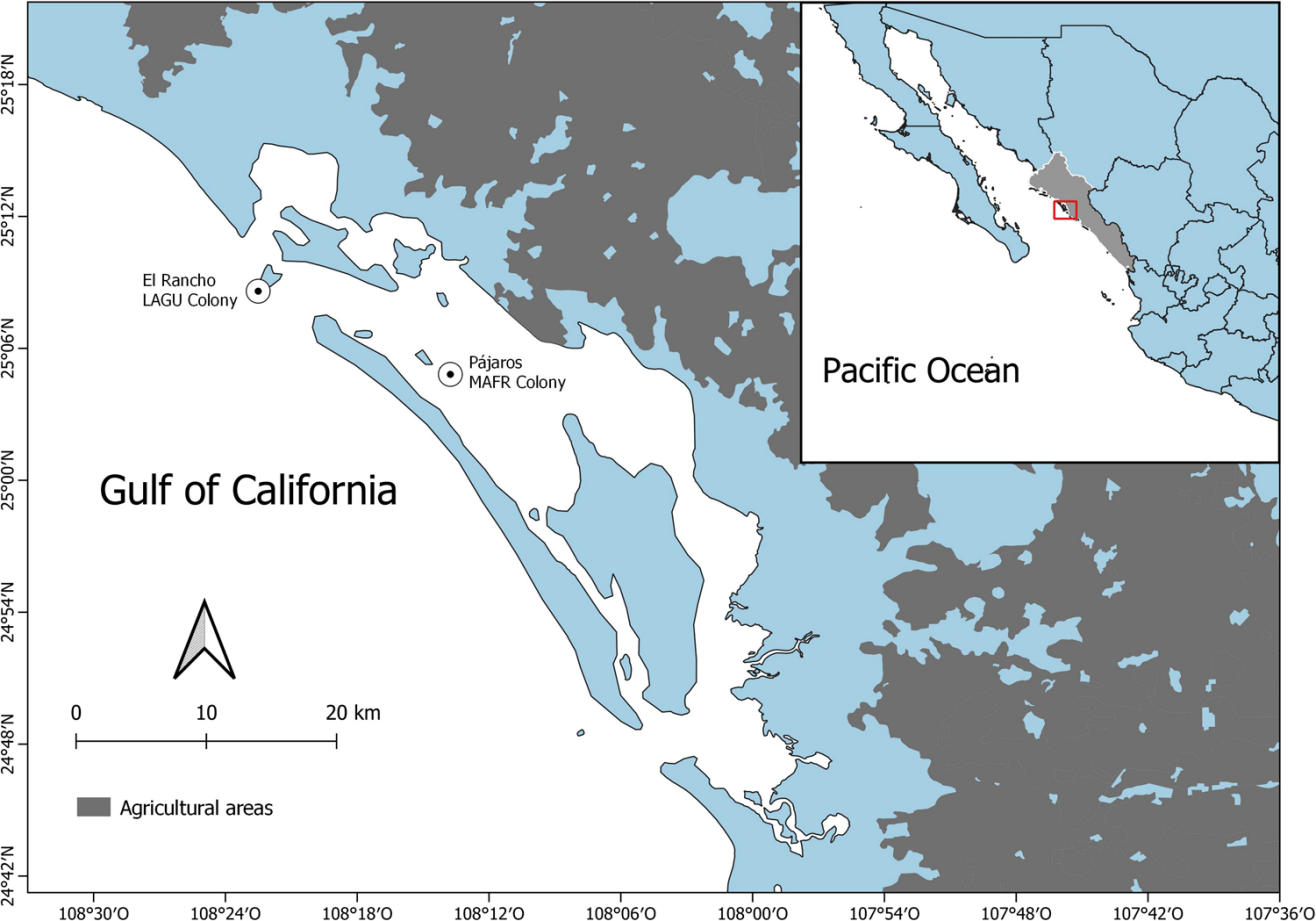
Geographic location of the study area: Bahía Santa María (BSM), south-central Gulf of California, Mexico. Shading represents agricultural areas. Locations of the Laughing Gull (LAGU) and Magnificent Frigatebird (MAFR) colonies are shown

Samples were collected from two islands in Bahía Santa María (BSM), located on the coast of Sinaloa near the southeastern region of the Gulf of California (Fig. 1). This is the largest coastal lagoon in Sinaloa. It is separated from the Gulf of California by Altamura Island, a long sandy bar spanning 45 km that protects the lagoon from wave action (Alvarez-Arellano and Gaitán 1994). The lagoon is surrounded by technified (131,243 ha) and rainfed (10,071 ha) agriculture, shrimp farms (7700 ha), and several human settlements (180,596 inhabitants; Páez-Osuna et al. 2007; INEGI (2010)). The continental waters that reach BSM primarily come from the Mocorito river basin and a vast network of agricultural drains that transport waste from aquacultural and farming activities and the cities of Culiacán and Guamúchil (Páez-Osuna et al. 2007; Acosta-Velázquez and Vázquez-Lule 2009; Montaño-Ley and Páez-Osuna 2014).

El Rancho (25° 09′ 14” N, 108° 22′ 19” W; 380 ha; Fig. 1) is a barrier island located in the northern region of the coastal lagoon. This island hosts approximately 22,000 Laughing Gull pairs, making it the largest breeding colony on the western coast of Mexico (Castillo-Guerrero et al. 2014). Located in the central region of BSM, Pájaros Island (25° 04′ 44” N, 108° 13′ 52” W; Fig. 1) is covered by mangrove forests and harbors approximately 8,000 Magnificent Frigatebird pairs (Castillo-Guerrero et al. 2014). At El Rancho, samples from 128 Laughing Gull individuals were collected between May and June 2011 (stable isotope analyses = 72 samples; trace elements analysis = 53 samples; organochlorine pesticide analysis = 54 samples). At Pájaros Island, 86 Magnificent Frigatebird individuals were sampled between January and April 2011 (stable isotope analyses = 66 samples; trace elements analysis = 68 samples; organochlorine pesticide analysis = 26 samples). Details of the sample sizes according to sex and breeding stage are described in Table S1; information on the simultaneous samples that were collected is provided in Table S2. Prey samples were also collected when the birds regurgitated spontaneously when being handled or when we walked through the colony; prey that exhibited minimal digestion were used as reference samples for isotope analysis.

The birds were captured directly at the nest, and blood samples were obtained by brachial vein puncture with a syringe (3 mL, 25 G, 0.5 mm × 16 mm). The collected blood was deposited in 1.5 mL plastic tubes (1.0 mL for stable isotope or trace elements analysis) or 5-mL vacutainers (for organochlorine pesticide analysis). To extract organochlorine pesticides, tubes were prepared with EDTA K2 (0.25 M, 100 μL), and the blood was centrifuged at 2700 × *g* for 15 min. The whole-blood samples (for isotopic and trace elements analyses) and the plasma obtained from centrifugation (for organochlorine pesticide analysis) were stored at –18 °C until use in the laboratory. Before being released, the birds were weighed, and sex, breeding stage, and morphometric measurements of the tarsus, ulna, and culmen were recorded.

The Magnificent Frigatebird exhibits sexual dimorphism in size and plumage (Diamond and Schreiber 2020). Hence, sex was identified unequivocally. Laughing Gull sex was determined based on culmen length (LC), total body length (TL), and wing length (W) using the discriminant function of Evans et al. (1993):

1 $$X=0.649{LC}+0.579{TL}+0.762W.$$

We employed a classification value of *X* = 507.392; individuals with *X* values greater than the classification value were identified as males, whereas those with *X* values below the classification value were identified as females. We verified the assigned sex by comparing the sizes of both parents when they were side by side and by observing copulation. Identification was correct in 97.3% of cases (two misclassified females were correctly identified before conducting any analysis). Based on their breeding stage, the sampled birds were categorized as being in either incubation or chick-rearing phases. We also focused on nests containing small chicks, defined as those less than two weeks old for the Laughing Gull and less than four weeks old for the Magnificent Frigatebird.

### Hg, Cd, and Pb analyses

The blood samples for non-essential trace elements analyses (1–1.5 mL) were lyophilized for 72 h and homogenized in a mortar. The homogenized samples were weighed (0.25 g) and placed in a digestion vessel with 5 mL of nitric acid and 3 ml of hydrogen peroxide. For digestion, the samples were placed in a Mars X microwave oven (CEM Corporation, Matthews, USA). Subsequently, a second digestion was conducted, in which 3 mL of hydrogen peroxide was added to each vessel. Once the samples were digested, they were diluted to 50 mL with distilled water.

The digested samples were analyzed by anodic stripping voltammetry (797 VA Computrace; Metrohm, Herisau, Switzerland). The concentrations of Cd and Pb were determined simultaneously using a rotating platinum electrode, a platinum auxiliary electrode, and a reference electrode (METROHM (2023a)). The Hg concentrations were determined with a rotating gold electrode (Au RDE), a glassy carbon auxiliary electrode, and a reference electrode (METROHM (2023b)). A blank, a certified reference material for trace metals (DOLT-5 Dogfish Liver) from the National Research Council of Canada, and a duplicate were included in each batch of 10 samples.

The detection limit for Cd and Pb was 0.1 mg/kg, whereas the detection limit for Hg was 0.2 mg/kg. The concentrations of Hg, Cd, and Pb were calculated using ppm dry weight and then converted to wet weight using the moisture content of the sample to facilitate comparisons with the literature. The recovery percentages for Pb, Cd, and Hg were 76, 77, and 95%, respectively, and relative percent differences between duplicated samples of 0, 3.7, and 0.12 were obtained. The samples were analyzed at the CIAD Environmental Sciences Laboratory in Guaymas, Sonora.

### Organochlorine pesticide extraction and analyses

The analysis of organochlorine pesticides was performed at the Laboratory of Chromatography, CIAD-Mazatlán, Sinaloa (Mexico). The analytical protocol followed the methodology of Matos-Lino et al. (1998) with modifications and involved processing 1 mL of plasma by liquid-liquid extraction with clean-up on silica gel. This allowed for the analysis of 19 organochlorine pesticides: aldrin, dieldrin, endrin, endrin aldehyde, endrin ketone, α- and β-endosulfan, endosulfan sulfate, heptachlor, heptachlor epoxide, α-, β-, γ- and δ-hexachlorocyclohexane (HCH), trans- and cis-chlordane, 4,4′-dichlorodiphenyldichloroethylene (4,4´-DDE), 4,4′-dichlorodiphenyldichloroethane (4,4´-DDD), and 4,4′-dichlorodiphenyltrichloroethane (4,4´-DDT). Internal standards were used to quantify the compounds using gas chromatography (Hewlett-Packard 5890 Series II, Palo Alto, USA) equipped with electron capture detectors. Most organochlorine pesticides were separated using Rtx-CLPesticides and Rtx-CLPesticides 2 columns (30 m × 0.25 cm × 0.2 μm; Restek Corporation, Bellefonte, USA) with nitrogen as the carrier and auxiliary gas. The detection limits of the organochlorine pesticides analyzed in plasma were between 7.5 and 7.8 ng/mL, and the quantification limits were between 22.5 and 23.3 ng/mL. Plasma concentrations were corrected for average procedural blank values. More details on the organochlorine pesticide extraction and analysis methodology can be found in the supplementary material.

### Sample preparation and stable isotope analysis

Isotopic values (*δ*^15^N and *δ*^13^C values) were measured in whole blood from seabirds and fish muscle (from below the beginning of the dorsal fin) from regurgitated prey. Blood (0.3–0.5 mL) and muscle samples (0.5–0.7 mg) were dried in an oven at 60 °C for 24–48 h and ground into a fine powder. The dried samples were weighed and wrapped in tin capsules. Weights ranged from 700 to 1000 μg. The samples were subsequently sent to the Stable Isotope Facility at the Universidad Nacional Autónoma de México for *δ*^13^C and *δ*^15^N analysis. Isotope ratios are reported using the standard notation:

2 $${\rm{\delta }}{\rm{sample}}=\left(\frac{{Rsample}}{{Rstandard}}-1\right)\times 1000,$$

where δsample is the isotopic ratio of the sample, and R is the ratio of the heavy to light isotopes (^13^C/^12^C or ^15^N/^14^N) in the sample relative to Vienna Pee Dee Belemnite or atmospheric nitrogen, respectively. Blood samples consistently exhibited low mass percentage ratios in C and N (C:N < 4.0) due to their low lipid content (Post et al. 2007). Consequently, lipid extraction was unnecessary (Bearhop et al. 2000b). However, the analyses of prey samples showed a mean C:N ratio > 3, and arithmetic normalization was performed to correct for differences in lipid content between samples (Post et al. 2007).

### Statistical analysis

#### Concentration and correlations between pollutant groups

We calculated total concentrations for each affinity group as follows: ƩDDTs (4,4´-DDE, 4,4´-DDD, and 4,4´-DDT), ƩHCHs (α-, β-, γ-, and δ-), ƩDrins (aldrin, dieldrin, endrin, endrin aldehyde, and endrin ketone), Ʃheptachlors (heptachlor and heptachlor epoxide), Ʃchlordanes (trans- and cis-chlordane), and Ʃendosulfans (α- and β-endosulfan and endosulfan sulfate). Given the magnitude of the variance of the organochlorine pesticide concentrations in the plasma (ng/mL; wet weight), we logarithmically transformed (base 10) the data of all groups (Zar 2010). As a notable portion of the data fell below the detection limits, we estimated the mean concentrations for Hg, Cd, Pb, and each organochlorine pesticide affinity group using imputation methods for censored data (ROS and Kaplan-Meier; Helsel 2005) when 50% of values (maximum) were below the limits of detection. We compared species using the Peto-Prentice test with the ‘NADA’ package in R (Lee (2022)). To evaluate the relationships among pollutants, we performed Pearson correlations (considering the Bonferroni correction for multiple comparisons) between all organochlorine pesticide groups (ƩDDT, ƩHCH, ƩDrins, ƩHeptachlor, ƩChlordane, and ƩEndosulfan), Hg, Cd, and Pb for each species.

#### Stable isotope analysis

We conducted general linear models (GLM) to evaluate *δ*^13^C and *δ*^15^N separately for each species. Sex and breeding stage were factors, and the sampling date (days elapsed since the approximate start of the breeding season) was used as a continuous predictor. A set of competing models was built by considering such a base model, considering all variables and the interaction between sex and breeding stage. The model selection used the corrected Akaike information criteria (AIC_C_) and the corresponding AIC_C_ weights (Johnson and Omland 2004). Linear models were conducted in R version 4.4.2 (R Core Team, 2024) with additional functions provided by the R packages lme4 (Bates et al. 2015) and MuMIn (Barton 2024).

The MixSIAR model was used to calculate the fractional contribution of prey for each species (see Table S3 for details about prey isotopic signatures and sample sizes; see Figs. S1 and S2 for the isospace biplots in the supplementary material). This model estimates the contribution of sources (prey) to consumer tissues, considering the observed variability of source and mixture isotopic ratios, dietary isotopic fractionation, and elemental concentration. It uses a Bayesian approach to estimate the probability distributions of multiple sources (Parnell et al. 2008). Four prey items were considered for the Laughing Gull model (Pacific white shrimp [*Litopenaeus vannamei*], Cortez swimming crab [*Callinectes bellicosus*], Anchovy [*Anchoa* spp.], and Weevils [Curculionoidea]). In contrast, six prey items were used in the Magnificant Frigatebird model (Anchovy [*Anchoa* spp.], Pacific anchoveta [*Cetengraulis mysticetus*], Pacific thread herring [*Opisthonema libertate*], halfbeaks [Hemiramphidae], other fishes [species with similar isotopic signatures, including Mackerel {*Scomber* sp.}, Californian anchovy {*Engraulis mordax*}, Mackerel scad {*Decapterus macarellus*}, and Cortez swimming crab {*C. bellicosus*}]). Mean estimates of the fractional contribution ± SD with 95% Bayesian credibility intervals are reported. Whole-blood isotope discrimination factors derived from captive controlled studies of a piscivorous seabird were used to correct for tissue-diet trophic isotope fractionation (see review by Cherel et al., 2005; δ15N: 2.7 ± 0.4; *δ*^13^C: 0.0 ± 0.7). These values have already been used in other seabird studies (Raya Rey et al. 2012; González-Medina et al. 2017).

#### Accumulation patterns

Given that many pollutants were either undetected or detected in very low frequencies and the high correlations among some pollutants, our analyses focused on high-frequency and uncorrelated contaminants or groups to evaluate the influence of sex, reproductive stage, and trophic ecology on contaminant loads. For the Laughing Gull, we selected the sum of organochlorines (log ΣOCs) and Hg. For the Magnificent Frigatebird, we chose Hg, HCHs, and Drins (see the section “Concentrations and correlations between pollutant groups” for more details).

General linear models (GLMs) were used to analyze the concentrations of these primary contaminants separately. Before structuring the analyses, the potential covariates were evaluated to avoid collinearity. The *δ*^15^N and *δ*^13^C values in both species were related to the sampling date, so this variable was excluded. The GLMs of the contaminant concentrations (Hg and log ΣOCs [response variables] for the Laughing Gull, and Hg, HCHs, and Drins [response variables] for the Magnificent Frigatebird) were initially structured in a similar way for both species: sex and reproductive stage were treated as factors, whereas body mass, *δ*^13^C, and *δ*^15^N values were used as covariates. All GLMs were based on initial models that considered all variables and the interaction sex-breeding stage. Subsequently, a set of competing models was built by considering such a base model. The model selection used the corrected Akaike information criteria (AIC_C_) and the corresponding AIC_C_ weights (Johnson and Omland 2004). Model assumptions (constant variance and normality) were verified by visually inspecting quantile-quantile plots (Q-Q plot) and the plots of residuals against fitted values.

## Results

### Concentrations and correlations between pollutant groups

The most frequently detected contaminants in both species were Hg and ΣDrins (Table 1). In addition, ΣDDTs and ΣHCHs were detected in most of the samples collected from the Laughing Gull and Magnificent Frigatebird, respectively (Table 1). The Magnificent Frigatebird exhibited higher mean concentrations of Hg and ΣHCHs but lower concentrations of ΣDDTs than the Laughing Gull (Table 1). In contrast, Σheptachlors, methoxychlor, and Σendosulfans were not detected or were detected in very low frequencies (Table 1) and were not considered in the estimation and comparison of means between species. In both species, Cd and Pb were present in low concentrations and exhibited a low frequency of occurrence (Table 1). More details on trace elements and organochlorine pesticides for both species (grouped by sex) can be found in the supplementary material (Tables S4 and S5).

**Table 1:** Non-essential trace elements and organochlorine pesticides in the Laughing Gull and Magnificent Frigatebird breeding at Bahía Santa María (BSM), Mexico.

	Laughing Gull					Magnificent Frigatebird					Among species comparison	
	*n*	FO	Range	Mean	Median	*n*	FO	Range	Mean	Median	Chi	*P*
**Non-essential trace elements (mg/kg, whole blood)**												
Mercury (Hg)	53	43 (81%)	BDL-6.98	0.29		74	72 (97%)	BDL-7.46	1.13		62.8	**<0.001**
Cadmium (Cd)	53	12 (23%)	BDL-0.13	0.032	BDL	74	12 (16%)	BDL-0.21	0.029	BDL	1	0.3
Lead (Pb)	53	9 (17%)	BDL-0.32	0.12	BDL	74	16 (22%)	BDL-2.05	0.06	BDL	1.7	0.2
**Organochlorine pesticides (ng/mL, plasma)**												
4,4´- DDT	43	19 (44%)	BDL-52.22		BDL	33	0 (0%)	BDL		BDL		
4,4´- DDE	43	16 (37%)	BDL-44.84		BDL	33	10 (30%)	BDL-34.19		BDL		
4,4´- DDD	43	35 (81%)	BDL-129.74	14.30		33	0 (0%)	BDL		BDL		
ΣDDTs	43	37 (86%)	BDL-223.52	22.1		33	10 (30%)	BDL-34.19	5.42	BDL	16.9	**0.00004**
HCH-α	43	1 (2%)	BDL-0.82		BDL	33	9 (27%)	BDL-10.77		BDL		
HCH-β	43	3 (7%)	BDL-2.99		BDL	33	24 (73%)	BDL-102.59				
HCH-γ	43	5 (12%)	BDL-58.73		BDL	33	0 (0%)	BDL		BDL		
HCH-δ	43	15 (35%)	BDL-88.64		BDL	33	1 (3%)	BDL-0.59		BDL		
ΣHCHs	43	19 (44%)	BDL-88.64	10.4	BDL	33	25 (76%)	BDL-102.59	27.7		11.7	**0.0006**
Aldrin	43	6 (14%)	BDL-97.94		BDL	33	3 (9%)	BDL-11.49		BDL		
Dieldrin	43	42 (98%)	BDL-78.32	11.04		33	4 (12%)	BDL-15.08		BDL		
Endrin	43	2 (5%)	BDL-4.967		BDL	33	0 (0%)	BDL		BDL		
Endrin ketone	43	10 (23%)	BDL-53.71		BDL	33	24 (73%)	BDL-27.07				
Endrin aldehyde	43	1 (2%)	BDL-7.73		BDL	33	0 (0%)	BDL		BDL		
ΣDrins	43	42 (98%)	BDL-115.13	18.6		33	25 (76%)	BDL-31.11	9.0		0	0.9
Heptachlor	43	22 (51%)	BDL-125.75			33	1 (3%)	BDL-18.22		BDL		
Heptachlor epoxide	43	3 (7%)	BDL-336.72		BDL	33	0 (0%)	BDL		BDL		
ΣHeptachlors	43	22 (51%)	BDL-382.99	16.16		33	1 (3%)	BDL-18.22	—-	BDL		
Trans-Chlordane	43	11 (26%)	BDL-96.71		BDL	33	13 (39%)	BDL-50.65		BDL		
Cis- Chlordane	43	11 (26%)	BDL-58.39		BDL	33	1 (3%)	BDL-7.46		BDL		
ΣChlordanes	43	15 (35%)	117.18	11.2	BDL	33	13 (39%)	BDL-58.12	5.0	BDL	0	0.9
Endosulfan-α	43	0 (0%)	BDL		BDL	33	2 (6%)	BDL-8.76		BDL		
Endosulfan-β	43	0 (0%)	BDL		BDL	33	2 (6%)	BDL-17.07		BDL		
Endosulfan sulfate	43	0 (0%)	BDL		BDL	33	0 (0%)	BDL		BDL		
ΣEndosulfans	43	0 (0%)	BDL	—	BDL	33	4 (12%)	BDL-17.07	—	BDL		
Methoxychlor	43	0 (0%)	BDL	—	BDL	33	0 (0%)	BDL	—	BDL		
**Total OCPs**	**43**	**43 (100%)**	**1227**	**133**		**33**	**28 (85%)**	**BDL-336**	**111**			

*n* sample size, *FO* frequency of occurrence (the number/proportion of samples where a specific pollutant was detected), *Range* minimum-maximum values, *mean* mean concentration estimated by imputation methods for censored data, *BDL* Below detection limit

Chi and P are statistical values from comparisons between species with the Peto-Prentice test.

Multiple correlations were detected among the organochlorine pesticide concentrations in Laughing Gull blood (except Chlordanes vs Heptachlor; Table S6). In addition, log ΣOCs were correlated with all contaminant groups (Table S6). Thus, ΣOCs can serve as a general proxy for the concentration of all organochlorine pesticide groups. However, no correlation was found between the different organochlorine pesticide groups and trace elements (Table S6). In the case of the Magnificent Frigatebird, correlations were observed between ΣOCs and ΣDDTs, ΣHCHs, and Σchlordanes, while Σdrins, Σheptaclors, and trace elements were not correlated with other contaminants (Table S7).

### Stable isotope analysis (*δ*^13^C and *δ*^15^N)

The best-fitting model based on AICc values showed that sampling date and sex were the main predictors of the variation in isotopic signature for both species (Table 2). In the Laughing Gull, both *δ*^13^C and *δ*^15^N best-fitting models included sampling date, but *δ*^13^C also included sex (Table 2). In frigatebirds, the best-fitting model for *δ*^13^C included only the sampling date, whereas for *δ*^15^N included only sex (Table 2). Although, in each analysis, there was more than one model with similar performance (ΔAIC_C_ < 2), the sampling date, sex, and, to a lesser extent, the stage were included in all of these models (Table 2).

**Table 2:** Corrected Akaike Information Criterion values (AICc), and AICc increments (ΔAICc; in parentheses) of linear models to assess which factors explained variation in blood *δ*^13^C and *δ*^15^N in adult Laughing Gull and Magnificent Frigatebird

	Laughing Gull		Magnificent Frigatebird	
Model	*δ*^13^C	*δ*^15^N	*δ*^13^C	*δ*^15^N
Sampling date	234.69 (4.91)	**271.96 (0.00)**	**91.65 (0.00)**	161.15 (7.77)
Sex	248.21 (18.42)	331.29 (59.33)	102.31 (10.65)	**153.38 (0.00)**
Breeding stage	242.57 (12.78)	306.65 (34.69)	101.70 (10.05)	160.73 (7.35)
Sampling date + sex	**229.78 (0.00)**	273.13 (1.18)	93.81 (2.16)	155.33 (1.95)
Sampling date + stage	235.59 (5.81)	272.38 (0.42)	93.97 (2.31)	162.81 (9.43)
Sex + stage	236.08 (6.29)	306.68 (34.72)	104.04 (12.39)	153.49 (0.11)
Sampling date + sex + stage	230.06 (0.28)	273.37 (1.42)	96.25 (4.59)	154.91 (1.53)
Sampling date + sex + stage + sex*stage	232.38 (2.59)	275.12 (3.16)	95.03 (3.37)	156.34 (2.96)

Values in bold refer to the best-fitting models

**Fig. 2:**
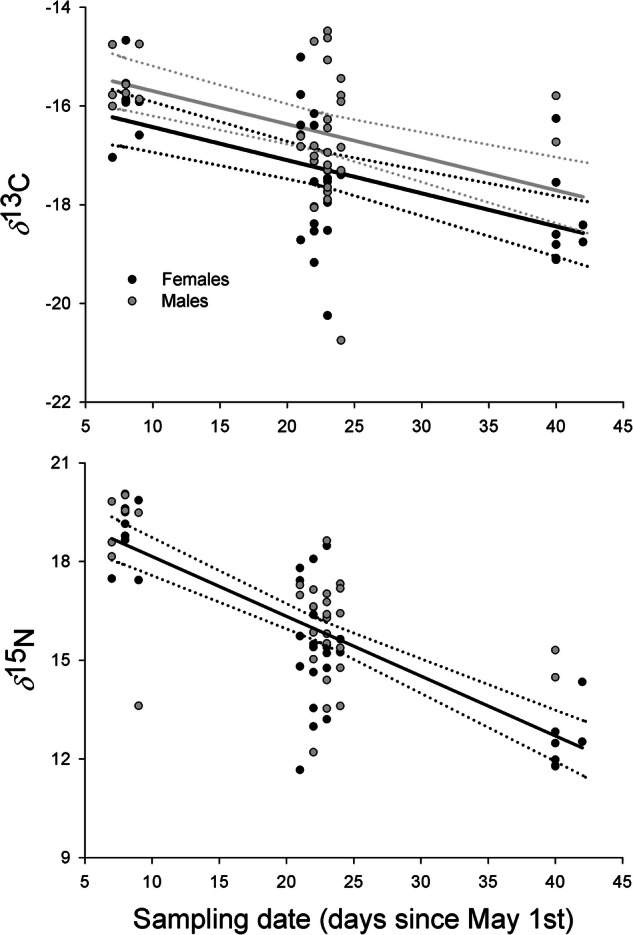
Whole-blood isotopic signatures (*δ*^13^C and *δ*^15^N) of the Laughing Gull as a function of sampling date and sex. The prediction and confidence interval of the best AIC-based model are shown

The isotopic signature of Laughing Gull blood underwent seasonal changes throughout the breeding season. Specifically, *δ*^13^C and *δ*^15^N decreased, and males showed *δ*^13^C values slightly higher than females (Fig. 2). Based on the results of the MixSIAR models, there was a notable increase in shrimp consumption towards the end of the breeding season. In contrast, the consumption of crabs and anchovies decreased (Table 3).

**Table 3:** Diet composition (%) of Laughing Gull adults during three periods

Dietary sources	Early May		Late May		Early June	
	Mean ± SD	95% IC	Mean ± SD	95% IC	Mean ± SD	95% IC
Pacific white shrimp	0.06 ± 0.13	0–48	0.47 ± 0.32	0–99	0.87 ± 0.2	18–100
Weevils	0.019 ± 0.08	0–16	0.11 ± 0.2	0–77	0.04 ± 0.13	0–44
Cortez swimming crab	0.74 ± 0.27	8–100	0.23 ± 0.25	0–86	0.06 ± 0.13	0–50
Anchovy	0.18 ± 0.24	0–83	0.18 ± 0.24	0–89	0.01 ± 0.06	0–13

Estimates based on *δ*^13^C and *δ*^15^N values of whole blood samples and prey species. Parameters were estimated using the Bayesian stable isotope mixing model MixSIAR. Mean estimates of the fractional contribution ± SD with 95% Bayesian credibility intervals are reported

**Fig. 3:**
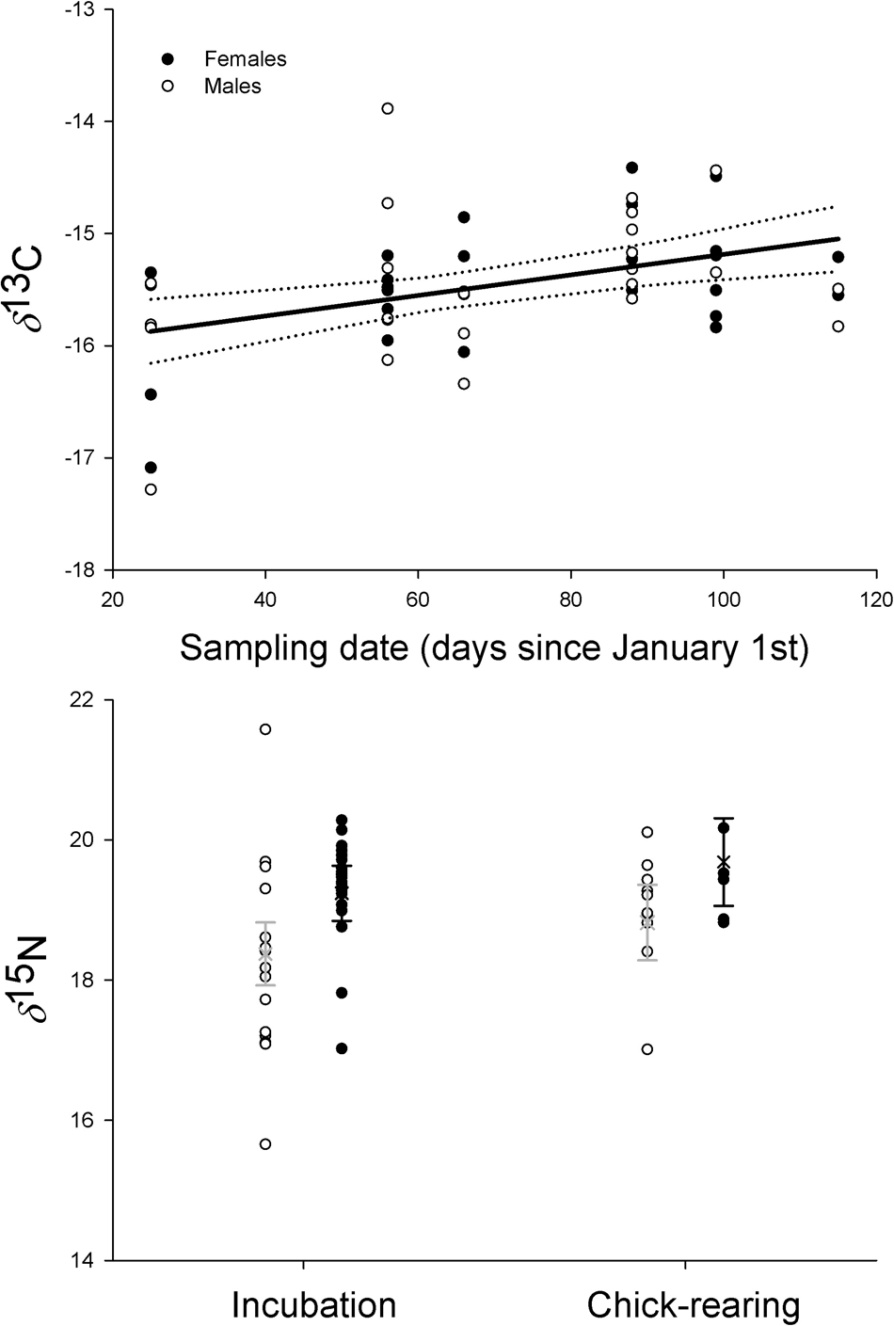
Whole-blood isotopic signatures (*δ*^13^C and *δ*^15^N) of the Magnificent Frigatebird as a function of sampling date and sex. The prediction and confidence interval of the best AIC-based model are shown

In Magnificent Frigatebird, *δ*^13^C values increased during the breeding season, and sex-related differences were observed, with higher *δ*^15^N values in females than in males (Fig. 3). Dietary consumption estimates revealed that females consumed more small pelagic fishes (Pacific thread herring and anchovy) and males consumed more crabs (Table 4).

**Table 4:** Diet composition (%) of Magnificent Frigatebird adults

Dietary source	Males		Females	
	Mean ± SD	95% IC	Mean ± SD	95% IC
Anchovy	0.08 ± 0.11	0–0.41	0.14 ± 0.13	0–0.45
Pacific anchoveta	0.19 ± 0.21	0–0.72	0.18 ± 0.15	0–0.54
Fishes (group)	0.06 ± 0.08	0–0.29	0.05 ± 0.06	0–0.23
Halfbeaks (Hemiramphidae)	0.15 ± 0.09	0–0.37	0.21 ± 0.09	0–0.30
Cortez swimming crab	0.45 ± 0.15	0.19–0.78	0.21 ± 0.09	0.07–0.41
Pacific thread herring	0.07 ± 0.09	0–0.36	0.21 ± 0.13	0–0.49

Estimates based on *δ*^13^C and *δ*^15^N values of whole-blood samples and prey species. Parameters were estimated using the Bayesian stable isotope mixing model MixSIAR. Mean estimates of the fractional contribution ± SD with 95% Bayesian credibility intervals are reported

### Accumulation patterns

**Fig. 4:**
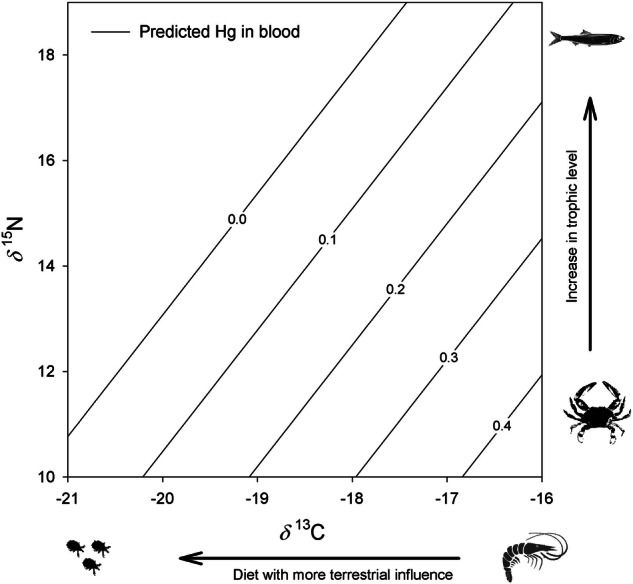
Expected Hg concentration in Laughing Gull whole blood (lines showing increases in 0.1 mg/kg concentration) according to the adjusted model based on *δ*^15^N and *δ*^13^C values. Model equation: Hg concentration in blood = 2.28 + 0.089* *δ*^13^C – 0.038* *δ*^15^N. Model multiple r^2^ = 0.15

**Fig. 5:**
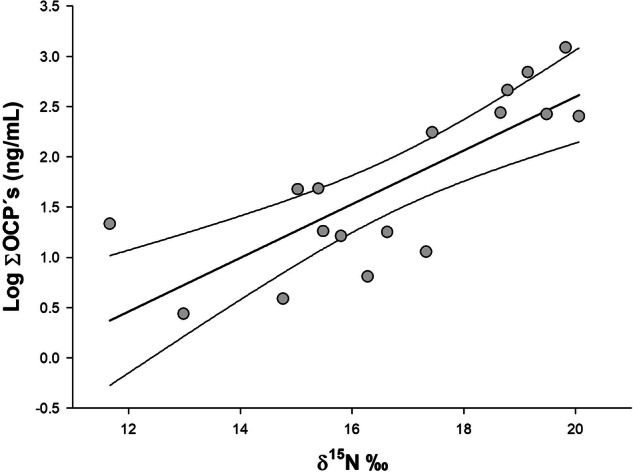
Concentration of OCs (logΣOC) related to *δ*^15^N in Laughing Gull plasma during the breeding season in Bahía Santa María (BSM), Mexico. The model-adjusted line ± standard error is shown

Using model information criteria for the Hg in the blood of Laughing Gull, the best-supported model included *δ*^13^C and *δ*^15^N as the main explanatory variables (Table 5). The Hg concentration in laughing gull blood was negatively related to *δ*^15^N and positively related to *δ*^13^C (Table 5; Fig. 4). The best-fitting model based on AIC_C_ values for the total concentration of organochlorine pesticides in Laughing Gulls included only the *δ*^15^N values (Table 6). In Laughing Gulls, the total concentration of organochlorine pesticides was positively related to *δ*^15^N (Fig. 5). The relationship between the isotopic signature and pollutants has an implicit temporal signal that was excluded from the model to avoid collinearity (both *δ*^13^C and *δ*^15^N decreased as the season progressed; see results of stable isotope analysis). The dynamics of blood contaminants in Magnificent Frigatebird did not exhibit a clear relationship to the variables analyzed. The best-fitting models for Hg, HCHs, and Drines included the null model, suggesting that the entire set of models has poor explanatory power (see detailed results in Tables S8–S10).

**Table 5:** Best candidate models evaluated to fit the data corresponding to Hg concentrations measured in the blood of Laughing Gull and their associated measures of information (AICc - corrected AIC; ΔAICc -AICc increments and AICc Wgt - AICc weights)

Intercept	*δ*^13^C	*δ*^15^N	body mass	stage	sex	sex* stage	AICc	ΔAICc	AICc Wgt
**2.2861**	**0.0890**	**−0.0386**					**4.74**	**0.00**	**0.27**
2.2616	0.0916	−0.0397	0.00028				7.18	2.43	0.08
2.2699	0.0901	−0.0369		+			7.18	2.44	0.08
2.2942	0.0896	−0.0382			+		7.21	2.46	0.08
0.9905	0.0488						7.43	2.68	0.07
0.1577							8.30	3.55	0.04
1.2072	0.0639			+			8.47	3.72	0.04
1.0837	0.0533				+		9.52	4.78	0.02
1.1056	0.0462		−0.00052				9.63	4.89	0.02
2.2437	0.0928	−0.0380	0.00029	+			9.73	4.99	0.02
2.2686	0.0932	−0.0393	0.00035		+		9.74	5.00	0.02
2.2783	0.0914	−0.0358		+	+		9.75	5.01	0.02
0.3446		−0.0118					9.95	5.21	0.02

Coefficient values for each model are shown. The most complete model included *δ*^13^C and *δ*^15^N signatures. The preferred model is highlighted in bold

**Table 6:** Best candidate models evaluated to fit the data corresponding to total organochlorines concentrations (Log ΣOCs) measured in plasma of Laughing Gull and their associated measures of information (AICc - corrected AIC; ΔAICc -AICc increments and AICc Wgt - AICc weights)

Intercept	δ^13^C	δ^15^N	body mass	sex	stage	sex* stage	AIC_C_	ΔAIC_C_	AIC_C_ Wgt
**−2.7476**		**0.2673**					**31.96**	**0.00**	**0.3362**
−2.8001		0.2792		+			33.20	1.24	0.1807
0.0825	0.1351	0.2329					33.94	1.98	0.1245
−2.8440		0.2528			+		35.02	3.05	0.0728
−2.1216		0.2786	−0.0026				35.08	3.11	0.0706
−0.2926	0.1195	0.2479		+			36.00	4.04	0.0445
−2.9397		0.2602		+	+		36.38	4.42	0.0368
−2.9397		0.2602		+	+	+	36.38	4.42	0.0368
−2.9773		0.2767	0.0007	+			37.30	5.33	0.0233
−0.1805	0.1256	0.2256			+		37.87	5.90	0.0175
0.1421	0.1264	0.2395	−0.0010				38.01	6.05	0.0162
−2.2469		0.2642	−0.0025		+		38.80	6.84	0.0109
−0.7591	0.1025	0.2374		+	+		40.36	8.39	0.0050
−0.7591	0.1025	0.2374		+	+	+	40.36	8.39	0.0050
−0.5372	0.1395	0.2334	0.0027	+			40.62	8.65	0.0044

Coefficient values for each model are shown. The most complete model included *δ*^15^N signature. The preferred model is highlighted in bold

## Discussion

The isotopic signatures, concentrations, and accumulation patterns of the contaminants in this study differed between species. The Magnificent Frigatebird exhibited higher mean concentrations of Hg and ΣHCHs but lower concentrations of ΣDDTs than the Laughing Gull. Stable isotope values exhibited a temporal pattern in isotopic signals (*δ*^15^N and *δ*^13^C in gulls, and *δ*^13^C in frigatebirds were associated with sampling date), and both species exhibited inter-sex differences (in *δ*^13^C for gulls and *δ*^15^N for frigatebirds). Finally, the Hg and ΣOCs concentrations in the Laughing Gull were related to isotopic values, but no pattern was detected in the Magnificent Frigatebird.

### Inter-species comparison of contaminant concentrations

The presence and concentrations of different pollutants varied between species, which was expected considering the interspecific differences in diet and habitat use. Considering that blood is generally helpful for assessing and monitoring recent pollutant exposure (Bearhop et al. 2000; Bustnes et al. 2004; Espín et al. 2016), the detected differences must correspond to the breeding period of each species. During this period, the diet of the Laughing Gull is broad. These seabirds feed in marine and inland habitats (up to 40 km inland; Dosch 2003) and interact with anthropogenic activities, such as agriculture (Burger 2020), while foraging. In contrast, the diet of the Magnificent Frigatebird is less varied (Sebastiano et al. 2017), with this seabird exclusively utilizing marine habitats, alternating between coastal and pelagic areas (up to 900 km; Austin et al. 2019). These differences in resource and area use can explain why several groups of organochlorine pesticides were more frequent and occurred in higher concentrations in the Laughing Gull despite its lower trophic level. In particular, foraging in coastal or inland areas likely increases pesticide exposure time and intensity. This is the case for the agricultural areas that surround BSM, where relatively high concentrations of organochlorine pesticides are present (García-de la Parra et al., (2012); Galindo-Reyes, Alegría (2018)) and constitute a direct source of pollutants for the Laughing Gull but not the Magnificent Frigatebird.

The differences between the trophic ecology of the Laughing Gull and the Magnificent Frigatebird include habitat use and trophic level. A previous study by Sebastiano et al. (2017) suggests that the lower diet diversity and higher trophic levels of the Magnificent Frigatebird compared to those of the Laughing Gull could explain the differences in Hg loads between these species. Unlike organochlorine pesticides, Hg occurs naturally in the environment and can increase in concentration and presence due to anthropogenic activities (Morel et al. 1998). The dominant Hg sources in the shallow ocean (50–150 m) are atmospheric deposition (Mason and Sheu 2002) and fluvial inputs (Liu et al. 2021), and ocean circulation patterns and different methylation zones can produce regional or local differences in bioavailability (Driscoll et al. 2013). Even if Hg and organochlorine pesticides are biomagnified within the food web, their different sources and spatial distributions could explain the distinct bioaccumulation patterns detected between species. As established in previous comparative studies, the interspecific differences reflect different exposure opportunities, diets, or even toxicokinetics (Burger et al. 2023).

### Toxicologic risk based on benchmarks

The mean Hg concentration in both species was within the threshold for moderate physiological effects and decreased reproductive success (0.2–1.0 μg·g^−1^ ww; Ackerman et al. 2016) and was within the range of values reported for these species in French Guiana (Laughing Gull: 0.5–5.8 μg·g^–1^ dw [0.1–1.2 μg·g^–1^ ww], Magnificent Frigatebird 3.8–7.8 μg·g^–1^ dw [0.8–1.6 μg·g^–1^ ww]; Sebastiano et al. 2016, 2017) and Delaware Bay, New Jersey (Laughing Gull: 0.47 μg·g^–1^ dw [0.059–0.990 μg·g^–1^ ww]; Burger et al. 2023). However, the organochlorine pesticide concentration was lower than the negative effect threshold or lethal level (Keith 1966; Greichus and Hannon 1973; Gress et al. 1973; Jehl 1973; Blus 1982; Peakall and Fox 1987; Elliott et al. 1988). Like those of other species in the region (e.g., Ceyca et al. 2016; Lerma et al. 2016; Piña-Ortiz et al. 2021), the Laughing Gull and Magnificent Frigatebird are exposed to trace elements and diverse organochlorine pesticides. No evident anomalies were observed during the breeding season nor in the survival of individuals, and the population estimates for the Laughing Gull and Magnificent Frigatebird were relatively stable in BSM. However, although the concentrations of some of these pollutants were relatively low, the possibility of interactive effects, which may be subtle and persist in the long term, remains.

The low concentrations of Cd and Pb in the blood of the studied species coincide with those reported in other regions, such as French Guiana (Sebastiano et al. 2016, 2017) and Delaware Bay (New Jersey; Burger et al. 2023). In the study area, similar low concentrations of these trace elements have been reported in the blood of seabirds, such as the Blue-footed Booby (Lerma et al. 2016), but relatively high concentrations of Cd were detected in the eggs of Laughing Gulls (BDL-2.7 μg/g, FO = 68%; Ceyca et al. 2016). In general, birds accumulate Cd through the food chain, initially in the kidneys and then in the liver and other organs (Lee 1996; Jin et al. 2012; Bahamonde et al. 2023). On the other hand, Pb is not metabolically regulated and accumulates mainly in the liver, kidneys, feathers, bones, and brain (Gochfeld et al. 1996; Jerez et al. 2011; Espejo et al. 2017; Bahamonde et al. 2023). Both trace elements remain in the blood briefly before being excreted or deposited in tissues. The half-life of Pb in avian blood is approximately 13 days (Williams et al. 2017). Whilst not explicitly reported, experimental studies suggest that radio-labelled Cd (^109^Cd) can disappear from the bloodstream within 48 hours (Sell 1975); it can also be reduced by up to 70% after 30 days of administering a Cd-free diet following 90 days of administering a diet containing Cd (White and Finley 1978). Blood is a dynamic tissue in which trace elements remain only for relatively short periods and do not accumulate as in other organs. This explains why they are detected in low concentrations and frequency.

Trace element levels in the blood reflect recent dietary exposure, whereas those in the kidneys and liver, for example, indicate chronic exposure (Friberg et al. 1986). Blood levels correlate with liver and kidney levels both within and between bird species (García-Fernández et al. 1995; Wayland et al. 2001) and increase according to the dose in chronically exposed birds (White and Finley 1978; Świergosz and Kowalska 2000). This demonstrates that blood is a suitable tissue for assessing Cd or Pb exposure, although their concentrations are often below standard detection limits, which can be mitigated by employing highly sensitive analytical methods (Stoeppler and Brandt 1980; García-Fernández et al. 1995). In our study, Cd and Pb concentrations in the blood were below the detection limits, which might suggest low exposure. However, this does not rule out the possibility that birds are entirely free of trace element exposure. Using blood measurements, although useful for regular monitoring, has limitations, as does using any other kind of tissue. Thus, future research could benefit from including other biomarkers, such as metallothionein (Cd-binding protein) or delta-aminolevulinic-acid dehydratase (δ-ALAD), which is a Pb-sensitive enzyme (Wang et al. 2014; Williams et al. 2017). Furthermore, assessing these pollutants in eggshells, feathers, regurgitates, or fresh carcasses could provide a complete overview of the deposition and accumulation in seabirds (e.g., Ceyca et al. 2016; Albert et al. 2019; Thébault et al. 2021).

### Accumulation patterns related to trophic ecology

Stable isotope analysis allowed us to detect temporal variations in the diet of the Laughing Gull. Temporal variation in foraging habitat use and food consumption in the Laughing Gull has been previously reported (Bernhardt et al. 2010; Washburn et al. 2013). Switching feeding habitats is part of the feeding strategy of this species. It appears to be related to changes in prey abundance and availability (Washburn et al. 2013) or to differences in the nutritional requirements of chicks and adults (Pierotti and Annett 1991; Pons 1994; González‐Medina et al., (2020)). In this study, changes in the isotopic signatures of the Laughing Gull were related to the pollutant concentration in the blood. At the beginning of the breeding season, gulls mainly use marine areas and consume high trophic-level prey (higher values of *δ*^13^C and *δ*^15^N), shifting towards terrestrial areas and lower trophic-level prey as the season progresses. Thus, changes in foraging habitat and diet influence the Hg concentrations. This finding is consistent with several studies that have indicated that habitat use is related to elevated Hg accumulation in biota because some specific habitats tend to accumulate Hg, such as marine benthic habitats (Le Croizier et al. 2019; Romero-Romero et al. 2022). In turn, the total organochlorine content in plasma was directly related to *δ*^15^N (trophic level), which indicates biomagnification (e.g., Ruus et al. 2002; Ricca et al. 2008). Overall, our results highlight the influence of diet on contaminant concentrations in the Laughing Gull, which were also linked to various spatiotemporal factors.

Inter-sex differences in *δ*^13^C in gulls suggest differential habitat use by males and females, with greater use of marine habitats by males. This may be part of a mechanism to avoid competition linked to the slight sexual dimorphism (e.g. González-Solís et al. 2000) or to differences in nutrient requirements during breeding (Machovsky-Capuska et al. 2016). However, this slight inter-sex habitat use was not reflected in the differential accumulation of contaminants (neither Hg nor organochlorine content) that would make more vulnerable or exposed one sex over the other in the Laughing Gull.

The sex-based differences in the isotopic signatures of the Magnificent Frigatebird are related to differences in foraging habitats and prey items. In this study, females consumed prey in higher trophic levels and a greater proportion of small pelagic fishes and exhibited less variability in isotopic values than males. This finding aligns with recent studies highlighting the differences in habitat selection and use between Magnificent Frigatebird males and females (Austin et al. 2019; Giambalvo et al. 2022). These studies have indicated that males use foraging areas farther away from the colony than females, allowing males to exploit different food sources. However, in two previous studies, no intersexual differences in blood *δ*^13^C and *δ*^15^N (Sebastiano et al. 2016; Trefry and Diamond 2017), diet, or foraging areas (Trefry and Diamond 2017) were detected in the Magnificent Frigatebird. This suggests an environmentally mediated foraging strategy, likely linked to differences in local conditions between colonies (Gilmour et al. 2018).

In this study, despite sex-based differences in isotopic signatures in the Magnificent Frigatebird, distinct patterns of contaminant accumulation related to trophic position or the use of other areas were not detected between females and males. It is possible that the mixed use of pelagic and coastal habitats, coupled with the consumption of a wide variety of prey items and the use of both short and extensive movements, resulted in contaminant accumulation without an established isotopic or sex-based pattern. This contrasts with a previous study that found a positive correlation between Hg and *δ*^15^N (Sebastiano et al. 2016). Thus, as foraging opportunities and pollutant sources can vary greatly among colonies, no generalizable patterns of pollutant accumulation remain even within species. Also, the Magnificent Frigatebird uses more homogeneous and broader habitats than the Laughing Gull, making it difficult to pinpoint specific patterns related to trophic ecology. Distinct patterns in contaminant concentrations in the blood of the Laughing Gull and Magnificent Frigatebird underline the complex interactions between seabirds and their contaminated environments, reflecting variable ecological and biological influences on contaminant uptake and accumulation.

## Conclusions

Our results highlight the relationship between the foraging behavior of seabirds and their exposure to environmental contaminants. The observed differences between the Laughing Gull and Magnificent Frigatebird are due to diet (trophic level) and foraging habitat, which affect contaminant bioaccumulation patterns. These species-specific patterns help us understand the complex interplay between seabirds and their environment, underscoring the need for continued monitoring and conservation efforts. It is crucial to comprehensively incorporate other tools to study seabird ecology and susceptibility to environmental contaminants. For example, GPS data could help disentangle the interactions between habitat use and spatial movements to better understand contaminant dynamics. Seabirds must respond to energy demands, food availability, and environmental conditions during the breeding season to maximize their opportunities. Thus, how seabirds use resources, and the space surrounding their colonies exposes them to various pollutants. The risks and consequences of such exposure must be understood in order to establish adequate regulatory measures to conserve the biodiversity of these sites properly.

## Supplementary information

Electronic Supplementary Material

## Data Availability

Data will be provided upon request.
