# Supplementary material for: Interactions between contaminants and the trophic ecology of two seabirds in a coastal lagoon of the Gulf of California
Source: Ecotoxicology. 2025 Jan 13;34(4):522–38. doi: 10.1007/s10646-025-02853-4 (PMC12049387; doi:10.1007/s10646-025-02853-4)

**Electronic Supplementary Material**

**Interactions between contaminants and the trophic ecology of two seabirds in a coastal lagoon of the Gulf of California**

José Alfredo Castillo-Guerrero^a^, Erick González-Medina^b^, Alberto Piña-Ortiz^c^, Miguel Betancourt-Lozano^d^, Jaqueline García-Hernández^e^, Salvador Hernández-Vázquez^a^ and Guillermo Fernández^f*^

^a^ Departamento de Estudios para el Desarrollo Sustentable de la Zona Costera, Centro Universitario de la Costa Sur, Universidad de Guadalajara, Gómez Farías 82, San Patricio-Melaque, Municipio de Cihuatlán, Jalisco CP48980, México

^b^ Conservation Biology Research Group, Área de Zoología, Universidad de Extremadura, Badajoz España

^c^ Department of Animal Ecology and Systematics, Justus Liebig University (JLU) Giessen, Heinrich-BuffRing 26, 35392 Giessen, Germany.

^d^ Unidad Mazatlán en Acuicultura y Manejo Ambiental, Centro de Investigación en Alimentación y Desarrollo A.C., Mazatlán, Sinaloa CP82100, México

^e^ Unidad Guaymas en Aseguramiento de Calidad y Aprovechamiento Sustentable de Recursos Naturales. Centro de Investigación en Alimentación y Desarrollo A.C. Guaymas, Sonora CP 85480, Mexico

^f^ Unidad Académica Mazatlán, Instituto de Ciencias del Mar y Limnología, Universidad Nacional Autónoma de México, Mazatlán, Sinaloa, México

* Corresponding author: G. Fernández. Email: gfernandez@ola.icmyl.unam.mx (ORCID 0000-0002-8478-5575)

**Organochlorine pesticide extraction and analyses**

Liquid-liquid extraction of the pesticides in plasma was carried out following the methodology of Matos-Lino et al. (1998) with modifications. Briefly, the plasma samples (1 mL) were placed in 10-mL glass tubes with 0.2 g of Na_2_Cl and 2.5 mL of a 9:1 hexane:acetone solution. The tubes were stirred in a vortex mixer for 1 min and allowed to stand for 5 min. Each sample was separated into two phases. The upper phase was extracted with a pipette and placed in a 5-mL glass tube. Then, 2.5 mL of hexane:acetone solution was added to the remaining lower phase, and the steps were repeated.

The resulting upper phases were combined and concentrated by evaporation and rotation in a miniVAP® (Grabner Instruments, Vienna, Austria) with nitrogen flow. The resulting extract was resuspended in 1 mL of hexane. Subsequently, the samples were cleaned with silica gel (EPA 3630c; EPA 1996). The extract was transferred to a polypropylene column with a pre-prepared fiberglass lower plug. This plug contained 1 g of commercial sand (Sigma Aldrich®) and 1 g of activated silica gel mixed with hexane. After the extract was added, an additional 1 g of commercial sand was introduced. The extracts were then eluted using 10 mL of hexane, followed by 10 mL of a 1:1 hexane:dichloromethane solution. The final extracts were concentrated through evaporation and rotation in a miniVAP with nitrogen flow and finally resuspended in 200 μL of hexane.

The samples were analyzed by gas chromatography with a Hewlett-Packard 5890 Series II gas chromatograph (Palo Alto, USA) equipped with two electron capture detectors coupled to different columns (Restek Corporation, Bellefonte, USA; Rtx-CLPesticides [cat. #11141] and Rtx-CLPesticides 2 [cat. #11324]) measuring 30 m (0.25-cm diameter and 0.2-μm film thickness) that were joined with a Press-Tight Y-universal deactivated connector (Restek Corporation; cat. # 20405261) to a Rxi guard precolumn (Restek Corporation; cat. # 10039) measuring 5 m (0.32-mm diameter). Nitrogen was used as the carrier and auxiliary gas.

A total of 2 μL of the extract was injected in splitless mode. The temperature conditions of the injector and detectors were 290 and 300 °C, respectively. The initial oven temperature was 150 °C. Two consecutive temperature gradients were programmed: (1) 5 to 245 °C/min and (2) 10 to 310 °C/min for 5 min. A mixture of standard solutions (Restek Corporation; Organochlorine Pesticide Resolution Check Mix [cat. # 32454]) was used to identify the following organochlorine pesticides: aldrin; dieldrin; endrin; endrin aldehyde; endrin ketone; α- and β-endosulfan; endosulfan sulfate; heptachlor; heptachlor epoxide; α-, β-, γ- and δ-hexachlorocyclohexane (HCH); trans- and cis-chlordane; 4,4′ -dichlorodiphenyldichloroethylene (4,4´-DDE); 4,4′ -dichlorodiphenyldichloroethane (4,4´-DDD); and 4,4′ - dichlorodiphenyltrichloroethane (4,4´-DDT). The detection limits of the organochlorine pesticides analyzed in plasma were between 7.5 and 7.8 ng/mL, and the quantification limits were between 22.5 and 23.3 ng/mL. The plasma concentrations of the pesticides are expressed in ng/mL. All analyses were conducted in the CIAD Laboratory of Chromatography in Mazatlán, Sinaloa.

**Table S1**. Sample size according to sex and breeding stage for Magnificent Frigatebirds and Laughing Gulls breeding in Bahía Santa María, Mexico. SIA (Stable Isotopes Analysis), Hg (mercury), Cd (cadmium), Pb (lead), and OCs (Organochlorine pesticides).

| **Sex/Breeding stage** | **Samples** | **Courtship** | **Incubation** | **Chick rearing** |
| --- | --- | --- | --- | --- |
| Magnificent Frigatebird | | | | |
| Male | SIA | 2 | 16 | 13 |
| Female | SIA | 5 | 22 | 6 |
| Male | Hg, Cd, Pb | 2 | 17 | 13 |
| Female | Hg, Cd, Pb | 5 | 23 | 6 |
| Male | OCs | 1 | 8 | 6 |
| Female | OCs | 2 | 9 | 5 |
| Laughing Gull | | | | |
| Male | SIA | 12 | 21 |  |
| Female | SIA | 14 | 25 |  |
| Male | Hg, Cd, Pb | 9 | 15 |  |
| Female | Hg, Cd, Pb | 13 | 16 |  |
| Male | OCs | 11 | 16 |  |
| Female | OCs | 12 | 11 |  |

**Table S2**. Number of individuals with stable isotope (SIA), trace elements (mercury, cadmium, and lead), and organochlorine pesticides (OCs) sampled simultaneously (grouped by species).

| Simultaneus sampling | Magnificent Frigatebird | Laughing Gull |
| --- | --- | --- |
| Trace elements, OCs and SIA | 26 | 8 |
| Trace elements and SIA | 66 | 50 |
| OCs and SIA | 26 | 19 |

**Table S3**. δ^13^C and δ^15^N values (Mean +/- SD) and C:N ratio of the prey species of Laughing Gull (LG) and Magnificent Frigatebird (MF).

| Sources | δ^13^C | δ^15^N | C:N ratio | n | Sp. |
| --- | --- | --- | --- | --- | --- |
| Pacific anchoveta | -15.85 ± 1.3 | 17.47 ± 0.70 | 5.6 | 37 | MF |
| Anchovies | -16.59 ± 0.64 | 17.45 ± 0.66 | 3.4 | 29 | MF, LG |
| Pacific thread herring | -15.16 ± 1.6 | 17.85 ± 0.40 | 5.9 | 21 | MF |
| Pacific white shrimp | -17.30 ± 0.35 | 9.38 ± 0.88 | 3.5 | 14 | MF, LG |
| Beetles/woodlice | -20.17 ± 0.47 | 9.51 ±1.17 | 6.1 | 9 | LG |
| Longfin halfbeak | -16.84 ± 0.4 | 18.21 ± 0.76 | 3.4 | 7 | MF |
| Cortez swimming crab | -14.38 ± 0.59 | 13.41 ± 2.66 | 3.8 | 2 | MF, LG |
| Group:  Blue mackerel  Californian anchovy  Mackerel scad | -17.47 ± 0.9 | 17.59 ± 0.7 | 3.8 | 21 | MF |

|  | **Males** | | | | | | **Females** | | | | | |
| --- | --- | --- | --- | --- | --- | --- | --- | --- | --- | --- | --- | --- |
|  | **n** | **FO** | **Max** | **Mean** | **SD** | **Med** | **n** | **FO** | **Max** | **Mean** | **SD** | **Med** |
| **Trace elements (mg/kg, whole blood)** | | | | | | | | | | | | |
| Mercury (Hg) | 24 | 18 | 6.98 | 0.43 | 1.41 | 0.08 | 29 | 23 | 1.52 | 0.16 | 0.28 | 0.086 |
| Cadmium (Cd) | 24 | 6 | 0.11 | 0.03 | 0.03 | BDL | 29 | 6 | 0.13 | 0.03 | 0.03 | BDL |
| Lead (Pb) | 24 | 6 | 0.32 | 0.18 | 0.04 | BDL | 29 | 3 | 0.27 | 0.11 | 0.04 | BDL |
| **Organochlorine pesticides (ng/mL, plasma)** | | | | | | | | | | | | |
| 4,4´- DDT | 19 | 8 | 49.28 | 5.04 | 11.8 | BDL | 24 | 11 | 52.23 | 7.65 | 15.0 | BDL |
| 4,4´- DDE | 19 | 8 | 15.22 | 1.96 | 4.07 | BDL | 24 | 8 | 44.84 | 2.62 | 9.6 | BDL |
| 4,4´- DDD | 19 | 15 | 38.78 | 8.24 | 11.0 | 3.13 | 24 | 20 | 129.74 | 18.04 | 33.0 | 4.98 |
| ΣDDTs | 19 | 16 | 66.76 | 14.95 | 21.4 | 4.11 | 24 | 21 | 223.52 | 27.80 | 51.4 | 6.05 |
| HCH-α | 19 | 1 | 0.82 | -- | -- | BDL | 24 | 0 | -- | -- | -- | -- |
| HCH-β | 19 | 1 | 1.96 | -- | -- | BDL | 24 | 2 | 2.99 | -- | -- | BDL |
| HCH-γ | 19 | 3 | 58.73 | -- | -- | BDL | 24 | 2 | 33.57 | -- | -- | BDL |
| HCH-δ | 19 | 5 | 37.38 | 3.32 | 9.35 | BDL | 24 | 10 | 88.64 | 12.11 | 22.2 | BDL |
| ΣHCHs | 19 | 8 | 58.73 | 6.71 | 15.8 | BDL | 24 | 11 | 88.64 | 13.67 | 22.3 | BDL |
| Aldrin | 19 | 2 | 7.51 | -- | -- | BDL | 24 | 4 | 97.94 | -- | -- | BDL |
| Dieldrin | 19 | 18 | 51.43 | 9.72 | 15.2 | 3.98 | 24 | 24 | 78.32 | 11.95 |  | 3.16 |
| Endrin | 19 | 0 | -- | -- | -- | -- | 24 | 2 | 4.97 | -- | -- | BDL |
| Endrin ketone | 19 | 7 | 53.71 | 7.73 | 14.2 | BDL | 24 | 3 | 12.71 | -- | -- | BDL |
| Endrin aldehyde | 19 | 0 | -- | -- | -- | -- | 24 | 1 | 7.73 | -- | -- | BDL |
| ΣDrins | 19 | 18 | 105.15 | 16.68 | 29.3 | 4.59 | 24 | 24 | 115.13 | 20.04 |  | 4.18 |
| Heptachlor | 19 | 6 | 46.27 | 3.11 | 11.1 | BDL | 24 | 16 | 125.75 | 12.13 | 28.1 | 1.16 |
| Heptachlor epoxide | 19 | 1 | 336.73 | -- | -- | BDL | 24 | 2 | 5.22 | -- | -- | BDL |
| ΣHeptachlors | 19 | 6 | 382.99 | 20.83 | 93.5 | BDL | 24 | 16 | 125.75 | 12.55 | 28.5 | 1.16 |
| Trans-Chlordane | 19 | 2 | 8.86 | -- | -- | BDL | 24 | 9 | 96.71 | 9.18 | 23.7 | BDL |
| Cis- Chlordane | 19 | 4 | 7.64 | 1.27 | 2.51 | BDL | 24 | 7 | 58.39 | 19.12 | 12.26 | BDL |
| ΣChlordanes | 19 | 5 | 9.29 | 2.11 | 3.60 | BDL | 24 | 10 | 117.18 | 18.61 | 35.3 | BDL |
| Endosulfan-α | 19 | 0 | -- | -- | -- | -- | 24 | 0 | -- | -- | -- | -- |
| Endosulfan-β | 19 | 0 | -- | -- | -- | -- | 24 | 0 | -- | -- | -- | -- |
| Endosulfan sulfate | 19 | 0 | -- | -- | -- | -- | 24 | 0 | -- | -- | -- | -- |
| ΣEndosulfans | 19 | 0 | -- | -- | -- | -- | 24 | 0 | -- | -- | -- | -- |
| Methoxychlor | 19 | 0 | -- | -- | -- | -- | 24 | 0 | -- | -- | -- | -- |
| **Total OCPs** | **19** | **19** | **1227** | **120.78** | **278** | **26.67** | **24** | **24** | **1204** | **182.84** | **313** | **25.63** |

**Table S4.** Trace elements and organochlorine pesticides in Laughing Gulls (grouped by sex) breeding at Bahía Santa María, Mexico. n = sample size; FO = frequency of occurrence (the number of samples where a specific pollutant was detected); Max = maximum value; mean = mean concentration estimated by imputation methods for censored data; SD = standard deviation; Med = median; BDL = below detection limit.

**Table S5.** Trace elements and organochlorine pesticides in Magnificent Frigatebirds (grouped by sex) breeding at Bahía Santa María (BSM), Mexico. N = sample size; FO = frequency of occurrence (the number of samples where a specific pollutant was detected); Max = maximum value; mean = mean concentration estimated by imputation methods for censored data; SD = standard deviation; Med = median; BDL = Below detection limit.

|  | **Males** | | | | | | **Females** | | | | | |
| --- | --- | --- | --- | --- | --- | --- | --- | --- | --- | --- | --- | --- |
|  | **n** | **FO** | **Max** | **Mean** | **SD** | **Med** | **n** | **FO** | **Max** | **Mean** | **SD** | **Med** |
| **Trace elements (mg/kg, whole blood)** | | | | | | | | | | | | |
| Mercury (Hg) | 33 | 33 | 7.46 | 1.37 | 1.48 | 0.91 | 35 | 35 | 4.42 | 0.96 | 3.91 | 0.83 |
| Cadmium (Cd) | 33 | 4 | 0.07 | 0.02 | 0.01 | BDL | 35 | 7 | 0.21 | 0.04 | 0.04 | BDL |
| Lead (Pb) | 33 | 7 | 0.21 | 0.11 | 0.03 | BDL | 35 | 7 | 2.05 | 0.12 | 0.36 | BDL |
| **Organochlorine pesticides (ng/mL, plasma)** | | | | | | | | | | | | |
| 4,4´- DDT | 15 | 0 | -- | -- | -- | -- | 16 | 0 | -- | -- | -- | -- |
| 4,4´- DDE | 15 | 7 | 34.19 | 9.42 | 12.5 | BDL | 16 | 3 | 17.43 | 3.25 | 4.59 | BDL |
| 4,4´- DDD | 15 | 0 | -- | -- | -- | -- | 16 | 0 | -- | -- | -- | -- |
| ΣDDTs | 15 | 7 | 34.19 | 9.42 | 12.5 | BDL | 16 | 3 | 17.43 | 3.25 | 4.59 | BDL |
| HCH-α | 15 | 5 | 10.77 | 3.42 | 3.03 | BDL | 16 | 4 | 5.93 | 1.79 | 1.46 | BDL |
| HCH-β | 15 | 12 | 76.25 | 28.09 | 24.31 | 23.41 | 16 | 10 | 102.59 | 25.34 | 28.25 | 9.33 |
| HCH-γ | 15 | 0 | -- | -- | -- | -- | 16 | 0 | -- | -- | -- | -- |
| HCH-δ | 15 | 0 | -- | -- | -- | -- | 16 | 1 | 0.60 | -- | -- | BDL |
| ΣHCHs | 15 | 12 | 87.02 | 30.21 | 26.76 | 24.14 | 16 | 11 | 102.59 | 23.04 | 31.11 | 9.96 |
| Aldrin | 15 | 3 | 11.49 | 4.38 | 2.84 | BDL | 16 | 0 | -- | -- | -- | -- |
| Dieldrin | 15 | 1 | 15.08 | -- | -- | BDL | 16 | 3 | 8.41 | 2.53 | 1.86 | BDL |
| Endrin | 15 | 0 | -- | -- | -- | -- | 16 | 0 | -- | -- | -- | -- |
| Endrin ketone | 15 | 12 | 27.07 | 8.60 | 8.10 | 8.44 | 16 | 11 | 21.70 | 6.89 | 5.36 | 6.77 |
| Endrin aldehyde | 15 | 0 | -- | -- | -- | -- | 16 | 0 | -- | -- | -- | -- |
| ΣDrins | 15 | 12 | 31.11 | 11.19 | 11.10 | 9.48 | 16 | 12 | 21.70 | 7.55 | 5.40 | 7.94 |
| Heptachlor | 15 | 0 | -- | -- | -- | -- | 16 | 1 | 18.22 | -- | -- | BDL |
| Heptachlor epoxide | 15 | 0 | -- | -- | -- | -- | 16 | 0 | -- | -- | -- | -- |
| ΣHeptachlors | 15 | 0 | -- | -- | -- | -- | 16 | 1 | 18.22 | -- | -- | BDL |
| Trans-Chlordane | 15 | 4 | 50.66 | 8.18 | 13.42 | BDL | 16 | 7 | 17.70 | 3.01 | 5.86 | BDL |
| Cis- Chlordane | 15 | 1 | 7.46 | -- | -- | BDL | 16 | 0 | -- | -- | -- | -- |
| ΣChlordanes | 15 | 4 | 58.12 | 8.68 | 15.53 | BDL | 16 | 7 | 17.7 | 3.01 | 5.86 | BDL |
| Endosulfan-α | 15 | 2 | 8.77 | -- | -- | BDL | 16 | 0 | -- | -- | -- | -- |
| Endosulfan-β | 15 | 1 | 17.07 | -- | -- | BDL | 16 | 1 | 6.13 | -- | -- | BDL |
| Endosulfan sulfate | 15 | 0 | -- | -- | -- | -- | 16 | 0 | -- | -- | -- | -- |
| ΣEndosulfans | 15 | 3 | 17.07 | 3.13 | 5.05 | BDL | 16 | 1 | 6.13 | -- | -- | BDL |
| Methoxychlor | 15 | 0 | -- | -- | -- | -- | 16 | 0 | -- | -- | -- | -- |
| **Total OCPs** | **15** | **14** | **336.13** | **116.69** | **95.14** | **85.50** | **16** | **12** | **269.14** | **73.01** | **85.28** | **70.0** |

**Table S6.** Matrix of correlations between different pollutant groups in the blood of the Laughing Gull during the breeding season at Isla El Rancho, Sinaloa, Mexico. The correlation coefficient is shown at the top, and the P value is shown at the bottom. Significant correlations appear in bold.

|  | DDTs | HCHs | Drins | Heptaclors | Chlordanes | Log (∑OCP) | Hg | Cd | Pb |
| --- | --- | --- | --- | --- | --- | --- | --- | --- | --- |
| DDTs | – | **0.82** | **0.85** | **0.37** | **0.79** | **0.75** | 0.16 | - | - |
| HCHs | <0.001 | – | **0.80** | **0.61** | **0.77** | **0.79** | -0.37 | - | - |
| Drins | <0.001 | <0.001 | – | **0.59** | **0.65** | **0.80** | -0.27 | - | - |
| Heptaclors | 0.013 | <0.001 | <0.001 | – | 0.25 | **0.50** | -0.14 | - | - |
| Chlordanes | <0.001 | <0.001 | <0.001 | 0.1 | – | **0.67** | -0.37 | - | - |
| Log (∑OCP) | <0.001 | <0.001 | <0.001 | 0.001 | <0.001 | – | 0.03 | - | - |
| Hg | 0.69 | 0.36 | 0.51 | 0.73 | 0.36 | 0.94 | – | -0.05 | **0.33** |
| Cd | - | - | - | - | - | - | 0.73 | – | -0.18 |
| Pb | - | - | - | - | - | - | 0.018 | 0.18 | – |

**Table S7.** Matrix of correlations between different groups of pollutants in the blood of the Magnificent Frigatebird during the breeding season at Isla Pájaros, Sinaloa, Mexico. The correlation coefficient is shown at the top, and the P value is shown at the bottom. Significant correlations appear in bold.

|  | DDTs | HCHs | Drins | Heptaclors | Chlordanes | Log (∑OCP) | Hg | Cd | Pb |
| --- | --- | --- | --- | --- | --- | --- | --- | --- | --- |
| DDT’s | – | **0.50** | 0.09 | -0.02 | **0.42** | **0.62** | 0.16 | -0.17 | 0.23 |
| HCH’s | 0.009 | – | 0.12 | -0.08 | 0.30 | **0.86** | -0.06 | -0.25 | 0.29 |
| Drins | 0.72 | 0.53 | – | -0.07 | 0.08 | 0.33 | -0.20 | 0.30 | 0.34 |
| Heptaclors | 0.87 | 0.73 | 0.71 | – | -0.01 | 0.06 | -0.16 | -0.05 | -0.11 |
| Chlordanes | 0.04 | 0.18 | 0.70 | 0.96 | – | **0.48** | -0.06 | -0.14 | -0.12 |
| Log (∑OCP) | 0.001 | <0.001 | 0.12 | 0.74 | 0.02 | – | -0.07 | -0.05 | 0.32 |
| Hg | 0.47 | 0.81 | 0.36 | 0.46 | 0.78 | 0.76 | – | -0.10 | -0.18 |
| Cd | 0.38 | 0.25 | 0.17 | 0.78 | 0.64 | 0.82 | 0.64 | – | 0.31 |
| Pb | 0.34 | 0.15 | 0.12 | 0.60 | 0.51 | 0.11 | 0.42 | 0.16 | – |

**Table S8**. Candidate models evaluated to fit the data corresponding to Hg concentrations measured in the blood of Magnificent Frigatebird and their associated measures of information (AICc - corrected AIC; ΔAICc -AICc increments and AICc Wgt - AICc weights). Coefficient values for each model are shown. The best-fitting models (ΔAIC_C_ < 2) included the null model, suggesting that the entire set of models has poor explanatory power.

| Intercept | *δ*^13^C | *δ*^15^N | Body mass | stage | sex | sex*  stage | AIC_C_ | ΔAIC_C_ | AIC_C_ Wgt |
| --- | --- | --- | --- | --- | --- | --- | --- | --- | --- |
| 1.1294 |  |  |  | + |  |  | 205.85 | 0.00 | 0.13 |
| 1.1744 |  |  |  |  |  |  | 205.95 | 0.10 | 0.12 |
| 1.3492 |  |  |  |  | + |  | 206.85 | 0.99 | 0.08 |
| 2.2100 |  |  | -0.00078 |  |  |  | 207.31 | 1.45 | 0.06 |
| 4.3033 | 0.2046 |  |  | + |  |  | 207.40 | 1.54 | 0.06 |
| 3.4937 | 0.1499 |  |  |  |  |  | 207.75 | 1.89 | 0.05 |
| 1.2378 |  |  |  | + | + |  | 207.84 | 1.98 | 0.05 |
| 2.6451 |  | -0.0802 |  | + |  |  | 207.92 | 2.06 | 0.04 |
| 1.6191 |  |  | -0.00036 | + |  |  | 208.03 | 2.17 | 0.04 |
| 3.6940 | 0.1515 |  |  |  | + |  | 208.70 | 2.85 | 0.03 |
| 1.8132 |  | -0.0250 |  |  | + |  | 209.11 | 3.25 | 0.02 |
| 1.0938 |  |  | 0.00021 |  | + |  | 209.12 | 3.26 | 0.02 |
| 4.3538 | 0.1405 |  | -0.00076 |  |  |  | 209.23 | 3.37 | 0.02 |
| 4.3869 | 0.2031 |  |  | + | + |  | 209.48 | 3.62 | 0.02 |
| 2.8389 |  | -0.0392 | -0.00069 |  |  |  | 209.54 | 3.68 | 0.02 |

**Table S9**. Candidate models evaluated to fit the data corresponding to Hexacyclohexanes (∑HCHs) concentrations measured in the blood of Magnificent Frigatebird and their associated measures of information (AICc - corrected AIC; ΔAICc -AICc increments and AICc Wgt - AICc weights). Coefficient values for each model are shown. The best-fitting models (ΔAIC_C_ < 2) included the null model, suggesting that the entire set of models has poor explanatory power.

| Intercept | *δ*^13^C | *δ*^15^N | body mass | sex | stage | sex*  stage | AIC_C_ | ΔAIC_C_ | AIC_C_ Wgt |
| --- | --- | --- | --- | --- | --- | --- | --- | --- | --- |
| 30.82 |  |  |  |  |  |  | 252.73 | 0.00 | 0.21 |
| 77.32 |  |  | -0.0348 |  |  |  | 254.09 | 1.36 | 0.11 |
| -84.28 |  | 6.0692 |  |  |  |  | 254.37 | 1.64 | 0.09 |
| -86.00 |  | 9.8854 | -0.0529 |  |  |  | 254.61 | 1.88 | 0.08 |
| 27.35 |  |  |  | + |  |  | 254.91 | 2.18 | 0.07 |
| 18.44 |  |  |  |  | + |  | 255.15 | 2.41 | 0.06 |
| 67.82 | 2.3975 |  |  |  |  |  | 255.24 | 2.50 | 0.06 |
| 184.27 |  |  | -0.1049 | + |  |  | 255.94 | 3.21 | 0.04 |
| -134.87 |  | 8.4188 |  | + |  |  | 256.11 | 3.38 | 0.04 |
| 19.30 |  | 9.7096 | -0.1197 | + |  |  | 256.75 | 4.01 | 0.03 |
| 87.51 | 0.6940 |  | -0.0344 |  |  |  | 256.90 | 4.16 | 0.03 |
| -84.06 | 0.0113 | 6.0672 |  |  |  |  | 257.19 | 4.45 | 0.02 |
| -176.98 | -4.8315 | 11.1005 | -0.0579 |  |  |  | 257.45 | 4.72 | 0.02 |
| -85.67 |  | 5.3272 |  |  | + |  | 257.56 | 4.82 | 0.002 |
| 50.82 |  |  | -0.0232 |  | + |  | 257.68 | 4.94 | 0.002 |

**Table S10**. Candidate models evaluated to fit the data corresponding to Hexacyclohexanes (∑HCHs) concentrations measured in the blood of Magnificent Frigatebird and their associated measures of information (AICc - corrected AIC; ΔAICc -AICc increments and AICc Wgt - AICc weights). Coefficient values for each model are shown. The best-fitting models (ΔAIC_C_ < 2) included the null model, suggesting that the entire set of models has poor explanatory power.

| Intercept | *δ*^13^C | *δ*^15^N | body mass | sex | stage | sex*  stage | AIC_C_ | ΔAIC_C_ | AIC_C_ Wgt |
| --- | --- | --- | --- | --- | --- | --- | --- | --- | --- |
| 8.6184 |  |  |  |  |  |  | 191.23 | 0.00 | 0.25 |
| 46.6918 |  | -2.0074 |  |  |  |  | 192.71 | 1.48 | 0.12 |
| 19.8154 |  |  | -0.0083 |  |  |  | 193.05 | 1.82 | 0.10 |
| 7.1728 |  |  |  | + |  |  | 193.08 | 1.85 | 0.10 |
| -2.5901 | -0.7263 |  |  |  |  |  | 193.73 | 2.50 | 0.07 |
| 3.1590 |  |  |  |  | + |  | 193.92 | 2.69 | 0.06 |
| 38.7342 |  | -1.6379 |  | + |  |  | 195.24 | 4.01 | 0.03 |
| 46.5149 |  | -1.6159 | -0.0054 |  |  |  | 195.25 | 4.02 | 0.03 |
| 50.7230 |  | -2.4335 |  |  | + |  | 195.48 | 4.25 | 0.03 |
| 47.9691 | 0.0679 | -2.0195 |  |  |  |  | 195.53 | 4.29 | 0.03 |
| 2.5848 | -1.1740 |  | -0.0090 |  |  |  | 195.70 | 4.47 | 0.03 |
| -12.4238 | -1.2602 |  |  | + |  |  | 195.71 | 4.48 | 0.03 |
| 14.8830 |  |  | -0.0051 | + |  |  | 195.85 | 4.62 | 0.02 |
| 2.4977 |  |  |  | + | + |  | 196.66 | 5.43 | 0.02 |
| 10.2208 |  |  | -0.0050 |  | + |  | 196.74 | 5.51 | 0.02 |
| -14.8118 | -1.1870 |  |  |  | + |  | 196.85 | 5.62 | 0.01 |

**Figure S1**. Isospace representing carbon (*δ*^13^C) and nitrogen (*δ*^15^N) isotopic ratios obtained from Laughing Gull (whole blood) and the following prey samples: weevil beetles (whole animal), anchovy, Cortez swimming crab, and Pacific white shrimp (muscle tissue). Source data have been adjusted by discrimination means and SDs.


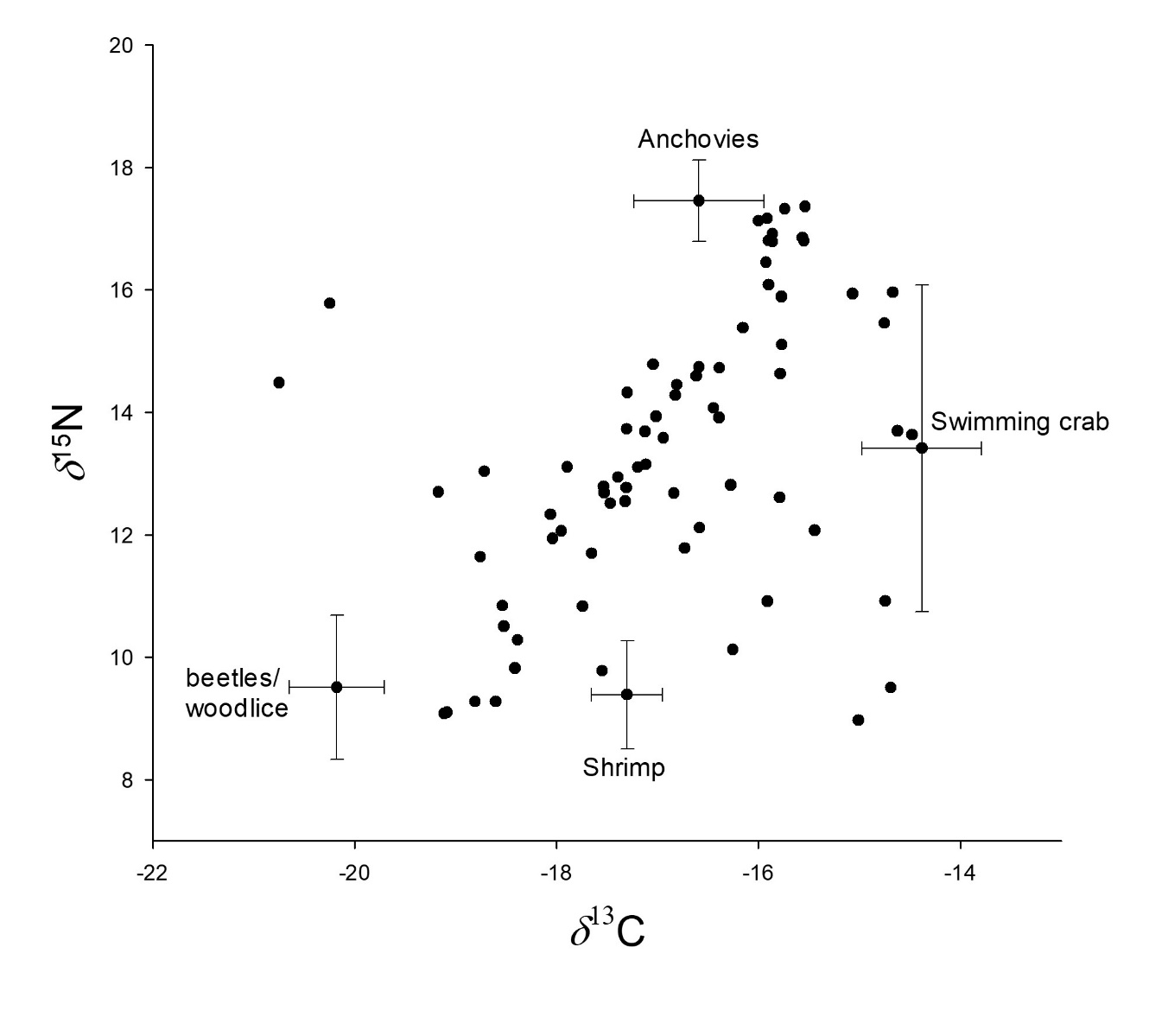


**Figure S2**. Isospace representing carbon (*δ*^13^C) and nitrogen (*δ*^15^N) isotopic ratios obtained from Magnificent Frigatebirds (whole blood) and prey samples (muscle tissue). Source data have been adjusted by discrimination means and SDs.


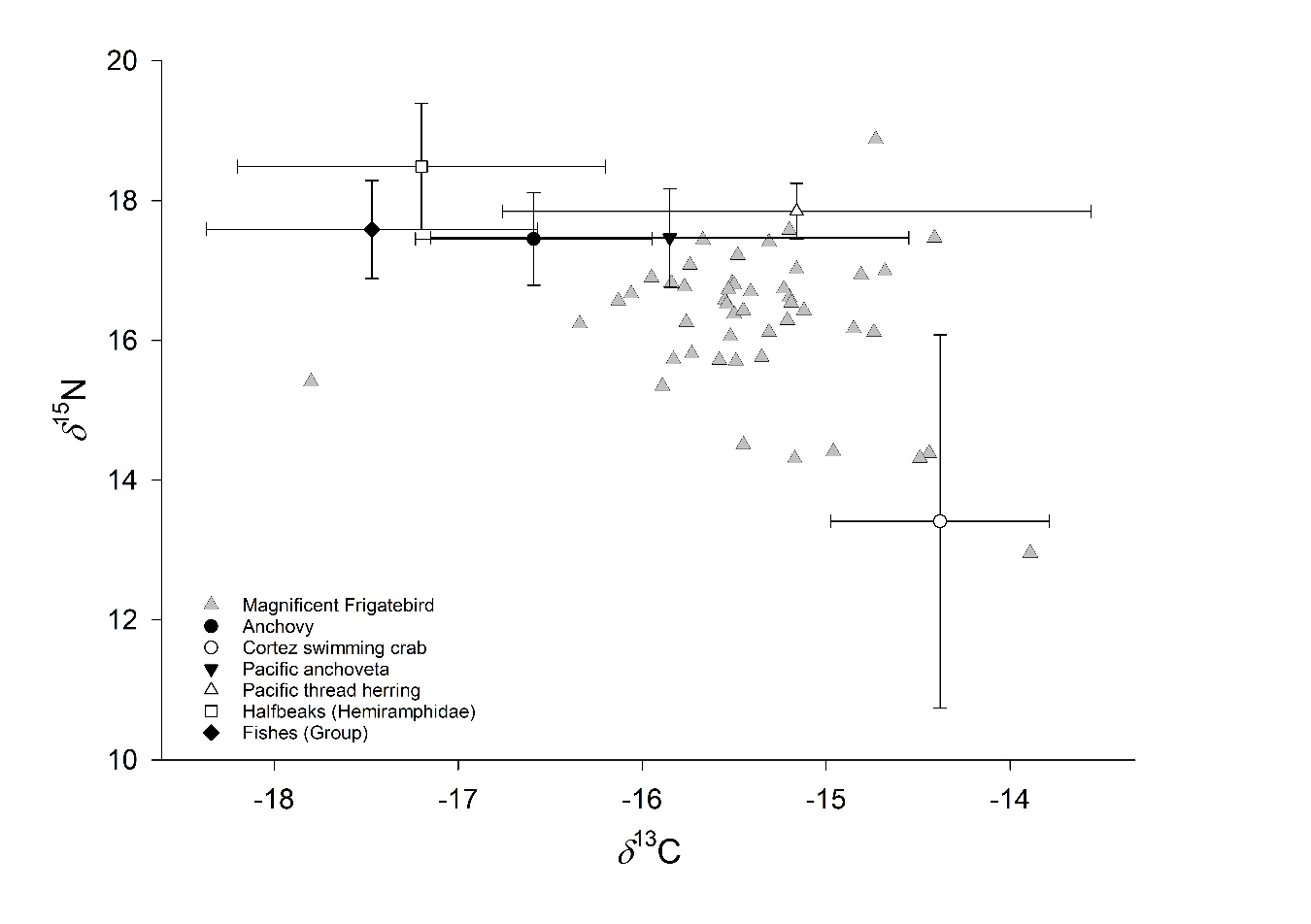

Supplement: Supplementary file 1 — Electronic Supplementary Material [file 10646_2025_2853_MOESM1_ESM.docx]
